# Trends in Epidemiology and Treatment of Humerus Fractures in the United States, 2017-2022

**DOI:** 10.7759/cureus.66936

**Published:** 2024-08-15

**Authors:** Kassem Ghayyad, Tyler F Beaudoin, Daryl C Osbahr, G. Russell Huffman, Amir R Kachooei

**Affiliations:** 1 Orthopedic Surgery, Rothman Orthopaedics at AdventHealth, Orlando, USA; 2 Orthopaedic Surgery, University of Maryland School of Medicine, Baltimore, USA; 3 Orthopaedic Surgery, Rothman Orthopaedics at AdventHealth, Orlando, USA; 4 Orthopaedics, University of Central Florida, Orlando, USA; 5 Orthopaedics, Rothman Orthopaedics at AdventHealth, Orlando, USA; 6 Orthopaedics, Orthopedic Research Center, Mashhad University of Medical Sciences, Mashhad, IRN

**Keywords:** current procedural terminology (cpt) code 24430, trinetx database, intramedullary nailing (imn), open reduction and internal fixation (orif), humerus shaft fracture, shaft of humerus fracture, diaphyseal shaft humerus fracture, humerus shaft fracture (hsf), humerus shaft fracture nonunion, humerus nonunion

## Abstract

Background

Fractures of the humerus are one of the more common fractures in the United States and a cause of fragility fractures in the elderly population. This study aims to understand recent trends in the demographic factors correlated with humeral shaft fractures (HSF) and humeral shaft fracture nonunion (HSFN) following open reduction internal fixation (ORIF) and intramedullary nailing (IMN).

Methods

The TriNetX database was used to query using International Classification of Diseases-10 (ICD10) diagnosis codes for patients who sustained HSF between 2017 and 2022. Patients were then organized into cohorts based on Current Procedural Terminology (CPT) codes 24515 and 24516 for ORIF and IMN of HSFs, respectively. Subsequent nonunion after operative management was queried. Descriptive and comparative analysis was performed to examine the differences observed between patients based on age, sex, ethnicity, race, and smoking status as well as surgical management across the six-year study period.

Results

The incidence of HSF increased from 7,108 in 2017 to 8,450 in 2022. The rate of HSF ORIF increased from 12% to 17% while the nonunion rate following ORIF decreased from 4% to 3%. The rate of HSF IMN increased from 4% to 6% and the rate of nonunion following IMN increased from 2% to 4%. The overall rate of HSFN surgery was 1.7% with slight decreasing trend over the past year.

Conclusion

It is speculated that improved care and surgical indications resulted in a lower rate of nonunion despite an increase in the overall rate of HSF and its operative managements.

## Introduction

Humeral shaft fracture (HSF) accounts for up to 6% of all fractures in the United States, leading to over 300,000 emergency room visits each year [1,2]. Additionally, HSF accounts for around 1-5% of all adult fractures managed in the United States each year, leading to significant morbidity and mortality for orthopedic patients [1,3-5]. HSF is generally thought to follow a bimodal age distribution, affecting both young and elderly patients, while decreasing in incidence during middle age [1,6]. These patients are generally either over 65 with a fragility fracture, or under the age of 30 suffering from a fracture secondary to high-energy trauma [2,6,7].

A major complication of HSF is nonunion, with some studies showing rates of nonunion following nonoperative fracture management of up to 30% [8-10]. Even among patients who undergo operative management of HSF, nonunion ranging from 4% up to 10% have been reported [8,11-14]. HSF nonunion (HSFN) can lead to significant disability in patients with decreased function, poor quality of life, and increased pain, which could potentially lead to dependence on narcotic pain control; as well as costing the healthcare system between $20,000 to $34,000 [5,11].

With rapidly changing demographic and population changes as well as a growing elderly population in the United States, fracture-related injuries are only expected to increase, leading to a need to better understand the impact these changes will have on HSF, especially those complicated by nonunion, to provide high-quality patient care. The purpose of this study is to utilize the TriNetX database, a global national collaborative network, to better understand the incidence and demographic factors underlying HSF and associated HSFN.

## Materials and methods

This was a retrospective study conducted in the United States in November 2023 using data from the TriNetX Global Collaborative Network database. The information from this database was de-identified; thus, it is exempt from the Institutional Review Board approval.

Patient cohorts were defined using the 10th edition of the International Classification of Diseases (ICD10) diagnostic codes. Patients with HSF (ICD-10: S42.301A, S42.302A) were queried from 2017 to 2022. Next, we used the Current Procedural Terminology (CPT) to find humerus fracture open reduction internal fixation (ORIF) (CPT:24515) and intramedullary nailing (IMN) (CPT:24516). Next, HSFN (ICD-10: S42.301K, S42.302K) was searched chronologically after humerus ORIF and IMN to find the nonunion rate after operative management of HSFs. CPT code 24430 related to humerus nonunion surgery was queried separately from 2017 to 2022 to see the overall nonunion surgeries after both operative and nonoperative management.

Demographical features

Patient information, including age, sex, ethnicity, race, and smoking status, was extracted. Age was divided into four quartiles: 0-17, 18-39, 40-64, and 65-90. Ethnicity was divided into Hispanic or Latino, Not Hispanic or Latino, and unknown. Race was divided into White, Black, Asian, Native American or Pacific Islander, American Indian or Alaska Native, and unknown.

TriNetX Global Collaborative Network

The Global Collaborative network through TriNetX is a web-based database tool allowing for population cohort research, feasibility queries, and collaboration with medical researchers worldwide. The database contains an extensive network of over 400 million de-identified patient data that can be accessed on demand without prior IRB approval. The database allows access to patient demographics, diagnoses, procedures, labs, and medications. Data are obtained through collaboration between over 200 community and academic-based healthcare organizations and industry partners worldwide. 

## Results

HSF demographics

We analyzed the frequency data of 46,578 HSFs from 2017 to 2022. The overall incidence of HSF was highest in the age groups of 40-64 and 65-90 years and lowest in the age groups of 0-17 and 18-39. HSF was more common in females (26,854 patients) than males (17,303 patients), with a female:male ratio of 1.6:1 (Table 1).

**Table 1:** Incidence rate of HSF and patient demographics Data are given as numbers unless indicated otherwise HSF: humeral shaft fracture

Year	2017	2018	2019	2020	2021	2022	2017-2022
Total HSF Patients	7,108	7,605	8,102	7,224	8,089	8,450	46,578
Age							
Mean± SD	55 ± 27	55 ± 27	55 ± 28	53 ± 28	52 ± 28	53 ± 28	43.8 ± 27.8
0-17 years	1053	1200	1353	1263	1528	1473	7870
18-39 years	1229	1337	1213	1236	1473	1396	7884
40-64 years	1403	1487	1631	1482	1677	1852	9532
65-90 years	3423	3581	3905	3243	3557	3729	21438
Sex							
Female	4095	4370	4739	4115	4637	4898	26854
Male	2959	3155	3292	3048	3373	1476	17303
Ethnicity							
Not Hispanic	4976	5454	5778	5295	5969	5919	33391
Hispanic	666	651	687	563	625	699	3891
Race							
White	5127	5532	5868	5267	6086	5781	33661
Black	806	793	850	848	905	904	5106
Asian	144	170	192	137	194	206	1043
Smoking History							
Yes	879	1031	1172	1108	1221	1277	6688
No	6,229	6,574	6,930	6,116	6,868	7,173	39,890

HSF ORIF

The overall rate of HSF ORIF from 2017 to 2022 was 15% with an increasing trend from 12% in 2017 to 17% in 2022 (Table 2).

**Table 2:** Distribution and rate of HSF ORIF patients (CPT: 24515) The percentages have been rounded to enhance clinical precision. HSF: humeral shaft fracture; ORIF: open reduction internal fixation; CPT: Current Procedural Terminology code

Year	2017	2018	2019	2020	2021	2022	2017-2022
Total HSF patients	7,108	7,605	8,102	7,224	8,089	8,450	46,578
Total HSF patients with ORIF	865	961	1,014	1,183	1,356	1,402	6,781
ORIF percentage	12%	13%	13%	16%	17%	17%	15%
Age							
Mean ± SD	52.5 ± 21.1	52.9 ± 21.3	53.1 ± 20.9	52.4 ± 21.3	50.7 ± 21.6	51 ± 22.2	52 ± 21.5
0-17 years	5	11	15	16	32	54	133
18-39 years	296	340	316	407	497	459	2315
40-64 years	286	269	312	331	371	402	1971
65-90 years	278	341	371	429	456	487	2362
Sex							
Female	405	459	497	600	724	674	3359
Male	406	444	530	451	599	601	3031
Unknown	54	58	66	47	83	77	385
Ethnicity							
Not Hispanic or Latino	620	719	719	880	995	1043	4976
Unknown	138	151	183	189	231	228	1120
Hispanic or Latino	107	91	112	108	130	131	679
Race							
White	586	645	699	791	895	914	4530
Unknown	119	124	141	133	179	168	864
Black	114	130	120	185	180	198	927
Asian	14	23	20	22	22	37	138
Native Hawaiian or other Pacific Islander	10	10	10	10	10	10	60
American Indian or Alaska Native	0	10	10	10	10	10	50
Smoking History (z87.891)							
Yes	154	180	192	200	206	233	1165
No	711	781	822	983	1,150	1,169	5,616

HSFN in ORIF

The overall rate of HSFN following ORIF was 3% with a slight decrease from 4% in 2017 to 3% in 2022 (Table 3).

**Table 3:** Demographics of patients with HSFN who underwent ORIF (CPT: S42.301K, S42.302K) The percentages have been rounded to enhance clinical precision. HSFN: humeral shaft fracture nonunion; CPT: Current Procedural Terminology code

Year	2017	2018	2019	2020	2021	2022	2017-2022
Total HSFN	865	961	1,014	1,183	1,356	1,402	6,781
Total HSFN with ORIF	32	36	45	30	36	49	228
HSFN with ORIF percentage	4%	4%	4%	3%	3%	3%	3%
Age (years), Mean ± SD	56.2 ± 16.2	58.3 ± 18.1	56.6 ± 21.4	55.8 ± 17.8	55.1 ± 22.3	48.9 ± 21.8	54.8 ± 20.1
Sex							
Female	19	17	26	15	21	26	124
Male	11	17	17	15	14	19	93
Unknown	10	10	10	0	10	10	50

HSF IMN

The overall rate of IMN after HSF was 5% with a slight increase from 4% in 2017 to 6% in 2022 (Table 4).

**Table 4:** Demographics and rates of HSF patients with IMN (CPT: 24516) The percentages have been rounded to enhance clinical precision. HSF: humeral shaft fracture; IMN: intramedullary nailing; CPT: Current Procedural Terminology code

Year	2017	2018	2019	2020	2021	2022	2017-2022
Total HSF Patients	7,108	7,605	8,102	7,224	8,089	8,450	46,578
Total HSF patients with IMN	273	293	372	397	430	474	2,239
IMN percentage	4%	4%	5%	5%	5%	6%	5%
Age							
Mean ± SD	55.4 ± 27.3	55.4 ± 27.4	54.9 ± 27.6	53.1 ± 27.9	52 ± 27.9	52.6 ± 27.5	43.8 ± 27.8
0-17 years	9	16	20	15	12	25	97
18-39 years	32	34	43	54	91	65	319
40-64 years	56	74	106	111	123	139	609
65-90 years	176	169	203	217	204	245	1214
Smoking History (z87.891)							
Yes	68	63	108	106	105	111	561
No	205	230	264	291	325	363	1,678

HSFN in IMN

The overall rate of nonunion following IMN of HSF was 2% with the highest rates in 2021-2022 (Table 5).

**Table 5:** HSF patients with nonunion after IMN (CPT: S42.301K, S42.302K) Exact numbers less than 10 could not be captured using the query. The percentages have been rounded to enhance clinical precision. HSF: humeral shaft fracture; IMN: intramedullary nailing; CPT: Current Procedural Terminology code

Year	2017	2018	2019	2020	2021	2022	2017-2022
Total HSF patients	865	961	1,014	1,183	1,356	1,402	6,781
Total HSFN patients with IMN	<10	<10	<10	<10	17	18	55
HSFN IMN percentage	N/A	N/A	N/A	N/A	4%	4%	2%

HSFN surgery

The humerus nonunion surgery rate among all of the humerus shaft fractures was 776 (1.7%) cases from 2017 to 2022 (Table 6).

**Table 6:** Humerus nonunion surgery (CPT: 24430) The percentage is calculated by dividing the number of nonunion surgeries by the total number of HSF HSF: humeral shaft fracture; CPT: Current Procedural Terminology code

	2017	2018	2019	2020	2021	2022	2017-2022
Total HSF patients	7,108	7,605	8,102	7,224	8,089	8,450	46,578
Total HSF patients with humerus nonunion surgery	121	137	159	117	119	123	776
Humerus nonunion surgery percentage	1.7%	1.8%	1.9%	1.6%	1.5%	1.5%	1.7%
Age							
Mean Age ± SD	62 ± 16	61 ± 18	60 ± 18	60 ± 16	61 ± 17	58 ± 20	60 ± 18
0-17	0	0	0	0	0	4	4
18-39	14	25	26	17	16	21	119
40-64	45	43	61	50	49	50	298
65-90	62	69	72	50	54	48	355
Sex							
Female	77	87	107	70	78	87	506
Male	44	47	52	47	41	36	267
Unknown	0	10	0	0	0	0	10
Ethnicity							0
Not Hispanic or Latino	98	105	120	83	93	97	596
Unknown	15	19	24	26	19	18	121
Hispanic or Latino	10	13	15	10	10	10	68
Race							0
White	106	115	129	94	96	94	634
Unknown	10	12	19	10	10	12	73
Black	10	10	10	10	10	10	60
Asian	10	10	10	10	10	10	60
Native Hawaiian or other Pacific Islander	10	0	0	10	10	0	30
American Indian or Alaska Native	10	10	10	10	10	10	60
Smoking Hx							0
Yes	38	37	56	33	38	33	235
No	83	100	103	84	81	90	541

## Discussion

Identifying demographic factors that underlie health outcomes can help better provide patient care, ensure proper patient counseling, and target interventions to improve health equity for at-risk groups. This study demonstrated numerous findings in incidence and demographic factors underlying HSF, HSFN following operative management, and the surgical management of humeral shaft nonunion. 

The vast majority of HSF, up to 70-80%, can be managed conservatively [5,15,16]. However, a study by Huttunen et al. saw a large increase in the number of patients treated surgically for HSF between 1987 and 2009, with rates of surgical management in males doubling to 46% and females tripling to 54% [17]. This trend, if it persists, could lead to a rise in surgical management of HSF overall, especially in older patients [18-20]. An increase in the rate of surgery might explain a decrease in the rate of nonunion rate, as primary surgical management may lower the rates of nonunion compared to nonoperative management [8].

There is no current universally accepted guideline on deciding between operative and nonoperative management of a patient with HSF, with the best treatment method still remaining controversial [21]. Sargeant et al. reported an 18% nonunion rate with nonoperative management compared to a 6.3% nonunion rate with fixation [22].

Although our study showed that the highest prevalence of HSF was among those over 65, the rates of nonunion as well as nonunion surgery were higher among middle-aged patients from 40-65. Several studies have looked at the trends in HSFN and age and have found that HSFN is much higher among middle-aged patients than the elderly, with rates of HSFN decreasing with increasing age [23,24]. A study by Zura et al. suggests that fracture in elderly patients is associated with premature death, which may lead to patients not surviving long enough for fracture nonunion to be diagnosed [23]. Elderly patients have lower rates of nonunion surgery, which could be attributed to patients being too high risk for surgical management or perceived worse outcomes post-surgical repair in this group due to delayed bone healing associated with age [25]. Studies have shown that surgical intervention for primary fractures has increased in elderly patients [26]. We speculate that it would lead to a lower nonunion rate as is implicated by our data. Similarly, this could be attributed to improvements in surgical techniques, as well as better initial outcomes with primary surgical repair in elderly patients who have worse bone healing and a higher risk for poor functional outcomes with nonoperative management [26-28].

Concerning gender, there was a higher prevalence of HSF among female patients as well as a higher rate of nonunion incidence when compared to males. However, both genders experienced similar rates of nonunion surgery. Numerous studies have shown that females have a much higher rate of HSF than men, at a ratio of around 2:1 [1,29]. This trend has been attributed to the higher prevalence of osteoporosis in elderly females, as well as females being more prone to low-energy traumatic events that could lead to HSF [30,31]. This may also partially explain the slightly higher rates of nonunion, as osteoporosis has been identified as a risk factor for delayed union and nonunion [32]. Another possible explanation for the higher nonunion rates in females is that males are more likely to experience an HSF from high-energy trauma which often requires immediate surgical intervention. Early, more definitive fixation leads to lower rates of nonunion. High energy trauma in the male cohort may also lead to outcomes such as loss of limb or death, meaning they may not live long enough to develop a nonunion [31]. Additionally, with males having more frequent HSF from trauma, the injuries may be more complicated and require primary surgical intervention, which has been shown to have lower rates of nonunion than non-operative management. However, the rate of nonunion surgery is similar between the two groups, indicating that while gender could be important at the time of initial injury, it is not observed in our data as an important factor in decision-making for nonunion surgery.

In a push to create a more equitable healthcare system, racial bias has come to light as a major barrier to equitable healthcare delivery and ensuring the highest outcomes for all groups of people [33]. In the United States, minority patient populations have been found to have worse health outcomes and less access to high-quality care compared to their white counterparts [34]. However, this study found no significant disparity between nonunion and nonunion surgical management among patients based on race or ethnicity. White patients had a higher prevalence of HSF, but all racial groups had comparable rates of ORIF and nonunion surgery. This follows the findings of other studies, which also displayed higher overall fracture rates in the white population, with a possible genetic component leading to higher fracture risk in white patients [35]. This trend is possibly attributed to higher rates of bone fragility and osteoporosis in white patients when compared to other racial groups. It may also explain the higher fracture rate and rate of nonunion in white patients [36]. While the study found a similar rate of operative fixation of HSF among all racial groups, we did not observe, as other studies have, that racial minorities receive less surgical intervention for fracture than white patients [35,37]. Numerous studies have shown that minority race and socioeconomic status are correlated with delayed operative repair and this delay in definitive treatment is a factor that requires exploration in future studies [35,38,39].

Another finding is that in 2019-2020, the start of the COVID-19 pandemic, the prevalence of HSF and HSFN decreased across the demographic groups. The decrease in the fracture rate is likely attributed to the lockdown resulting in lower rates of traumatic incidents in the vulnerable population [40-42]. However, the decrease in the rate of nonunion may be correlated with a higher rate of primary acute surgery resulting in a lower rate of nonunion, because the elective surgeries were replaced by the trauma surgeries to fill out the operating room block times. The rates of nonunion surgery were almost comparable over the years from 2017 to 2022 implying that care was provided and decisions were mainly driven by the symptoms rather than the nationwide situation [41].

The TriNetX database utilized in this study provides a heterogeneous set of patient data from 59 healthcare organizations across the country that is not otherwise available from a single institution. However, there are some notable limitations to this study. The dataset was only formed over five years and thus cannot be generalized for epidemiological trends over longer periods. The TriNetX dataset used in the study is formed directly from electronic health record (EHR) systems from multiple healthcare organizations and thus may have variability in the data received from these organizations. Additionally, the TriNetX database only includes data from select healthcare institutions and cannot account for patients who are diagnosed with and treated for HSF or HSFN at healthcare facilities that are not a part of the database. The database also does not include the mechanism of injury, fracture pattern, functional status, or outcome. Thus, this study cannot draw conclusions on indications or appropriateness of interventions. Additionally, the study does not account for patient factors such as insurance status, socioeconomic status, comorbidities, or geographic location.

The rate of nonunion showed a slight decrease with an increased number of ORIF while nonunion slightly increased with an increased number of IMN fixation. Appropriate indications for surgical intervention can mitigate the risk of nonunion, and nonunion surgery, its implications on the patients’ quality of life, and burdens on healthcare.

## Conclusions

The trend in the incidence of HSF only increased during the past six years and with an increase in the elderly population in the United States is only expected to continue to rise. However, rates of HSFN saw a decrease during the same period. Increased rates of surgical management with ORIF and IMN fixation for HSF likely underscores the decreasing rates of fracture nonunion observed, with relatively low rates of nonunion following both IMN fixation and ORIF. Improved initial care and increased surgical indications for HSF could result in lower HSFN even with fracture rates continuing to rise. Further studies on surgical indications for HSF could help clarify optimal fracture treatment and help reduce rates of HSFN. 
